# Severe maternal morbidity in patients of advanced maternal age with and without metabolic syndrome

**DOI:** 10.1007/s00404-026-08449-5

**Published:** 2026-05-05

**Authors:** Carolyn Robb, Rebecca Strafella, Jay Ayar, Olivia Prizzi, Su Htwe, Sarah Weiss, Elizabeth Drugge, Vani Dandolu

**Affiliations:** 1 New York Medical College School of Medicine, Valhalla, NY USA; 2 Lewis Katz School of Medicine at Temple University, Philadelphia, PA USA; 3 NYC Health and Hospitals/Metropolitan, New York, NY USA

**Keywords:** Advanced maternal age, Metabolic syndrome, National Inpatient Sample, Severe maternal morbidity

## Abstract

**Purpose:**

To estimate the burden of metabolic syndrome (MetS) on severe maternal morbidity (SMM) in women of advanced maternal age (AMA), quantify age-related escalation in SMM risk, and evaluate sociodemographic influences.

**Methods:**

We retrospectively conducted a cross-sectional, population-level analysis of the Nationwide Inpatient Sample (Q4 2015–2019), a discharge-level database, to identify delivery hospitalizations among patients aged ≥ 35 years. MetS was defined by ICD-10 codes indicating ≥ 3 of the following: hypertension, type 2 diabetes, obesity, hypertriglyceridemia, or low high-density lipoprotein cholesterol. The primary outcome was SMM as defined by the Centers for Disease Control and Prevention; a secondary outcome excluded blood transfusion. Multivariable logistic regression adjusted for race, income quartile, primary payer, hospital characteristics, and substance use. Weighted adjusted odds ratios (AORs) with 95% confidence intervals (CIs) were reported.

**Results:**

Among 3.1 million delivery hospitalizations, 556,609 (17.8%) occurred in AMA patients; 12,188 (2.2%) experienced SMM and 6,528 (1.2%) experienced nontransfusion SMM. SMM was significantly higher among patients with MetS compared to  those without (7.37% vs 2.17%; nontransfusion SMM: 5.36% vs 1.16%). SMM rates increased with advancing age. After adjustment, MetS was associated with markedly increased odds of SMM (AOR 2.75, 95% CI 2.29–3.30) and nontransfusion SMM (AOR 3.59, 95% CI 2.90–4.44), both p < 0.001.

**Conclusion:**

MetS is associated with SMM risk among AMA patients, independent of sociodemographic factors, with risk increasing further at older maternal ages. This study quantifies the disproportionate burden of MetS in this high-risk population.

## What does this study add to the clinical work?

**Table:** 

This study demonstrates that metabolic syndrome is the strongest independent predictor of severe maternal morbidity among patients of advanced maternal age, increasing the odds of severe maternal morbidity nearly threefold and exceeding the risk associated with age alone. These findings support incorporating preconception and prenatal screening for metabolic syndrome components into routine care for advanced maternal age patients to improve risk stratification, counseling, and referral for higher-level obstetric management.	

## Introduction

Severe maternal morbidity (SMM) refers to unexpected labor and delivery outcomes that result in significant short- or long-term health consequences [1]. The CDC defines SMM using 21 indicators, including blood transfusion, myocardial infarction, sepsis, and eclampsia [1]. SMM is multifactorial, with risk influenced by pre-existing chronic conditions, substance use, insurance status, geographic location, racial minority status, and maternal age [2]. As childbearing is increasingly delayed, understanding how maternal age and comorbidities influence SMM has become critical. Advanced maternal age (AMA), defined as ≥ 35 years at delivery, is associated with higher rates of maternal mortality and complications, such as miscarriage, preeclampsia, and cesarean delivery [3–5].

Chronic conditions like hypertension, type 2 diabetes, and obesity are more common in individuals of AMA and are known to increase SMM risk at delivery [6, 7]. For example, hypertensive disorders of pregnancy affect 18% of individuals aged 35–44 and 31% of those aged 45–55, while chronic hypertension alone has an overall prevalence of approximately 2% [8]. These disorders significantly increase the risk of SMM [9]. Similarly, type 2 diabetes and higher pre-pregnancy BMI, both more prevalent in AMA, are linked to greater pregnancy complications and SMM [10–14]. These comorbidities often co-occur and may act synergistically to heighten risk.

To evaluate their combined effect, we focused on metabolic syndrome (MetS), a cluster of conditions including hypertension, diabetes, and obesity [15]. MetS affects 33–42% of US adults, with prevalence increasing with age [16, 17]. While limited data exist for MetS in pregnancy and at delivery, global estimates suggest a prevalence of 16.3% [18]. A multicenter international prospective cohort study found that pregnant women with MetS had increased risks of pulmonary embolism and gestational diabetes mellitus, though maternal age was not evaluated [19]. Similarly, a prospective cohort study in Taiwan demonstrated that clustering of metabolic abnormalities among patients of AMA was associated with adverse pregnancy outcomes [20]. Because both AMA and metabolic comorbidities independently increase obstetric risk, examining their combined impact may provide important insight into SMM in this population. However, to our knowledge, no US population-level studies have examined the effect of MetS on SMM among patients with AMA. This study aims to address this gap by evaluating whether MetS is associated with increased risk of SMM in AMA and whether advancing maternal age and sociodemographic factors further modify this risk.

## Methods

This retrospective, cross-sectional, population-level study utilized data from the National Inpatient Sample (NIS) from Q4 2015 through 2019, a period selected to ensure exclusive use of ICD-10 coding [21]. The NIS is the largest all-payer inpatient database in the United States and provides nationally representative estimates of hospitalizations across a broad range of demographic and clinical populations [22]. Diagnostic and procedure codes were selected based on CDC guidelines and current clinical standards (Appendix 1). The study population included all hospitalized pregnant patients aged ≥ 35 years who delivered during the study period, identified using delivery-related ICD-10 codes (Appendix 1). Because the NIS is a discharge-level database without unique patient identifiers, hospitalizations cannot be linked across the same individual; therefore, antenatal admissions prior to delivery could not be captured, and this study was limited to delivery hospitalizations. The primary exposure was metabolic syndrome (MetS), defined by the presence of at least three of five components: obesity (ICD-10: E66.0–E66.2, E66.8, E66.9, Z68.3–Z68.4), hypertension (ICD-10: I10–I16*), type 2 diabetes mellitus (ICD-10: E11*), low HDL (ICD-10: E78.6, E88.1), and elevated triglycerides (ICD-10: E78.1–E78.5) [23]. To specifically capture baseline metabolic health, only chronic conditions were included in the definition of hypertension and diabetes. Pregnancy-related conditions, including gestational diabetes and hypertensive disorders of pregnancy, such as gestational hypertension and preeclampsia, were excluded, as these are often considered downstream manifestations of underlying metabolic dysfunction rather than pre-existing exposures. Pregnant individuals under age 35 were excluded.

The primary outcome was severe maternal morbidity (SMM), defined according to CDC criteria and ICD-10 codes (Appendix 1), among patients of advanced maternal age with and without MetS [1]. Patients with at least one qualifying SMM indicator were classified as experiencing SMM. We conducted analyses using two SMM definitions: one that incorporated blood transfusions and one that did not. Nontransfusion SMM was defined by the presence of one or more of the 20 CDC-recognized SMM indicators, excluding blood transfusions. Since blood transfusions account for over half of reported SMM cases and represent the leading contributor to overall SMM rates, their inclusion can inflate morbidity estimates [24]. Additionally, ICD-10 transfusion codes do not specify the number of units administered, potentially leading to overestimation by capturing cases with minimal transfusions that may not reflect clinically significant morbidity [24]. Moreover, compared to other SMM indicators, such as disseminated intravascular coagulation, hysterectomy, acute renal failure, and acute respiratory distress syndrome, blood transfusion is generally considered a less severe outcome. Evaluating nontransfusion SMM thus allows for focused analysis on the impact of MetS and other exposures on more severe morbidity outcomes among AMA patients.

Covariates included known risk factors for SMM in the NIS dataset including age subgroups (35–39, 40–44, ≥ 45), race, income quartile, insurance type, and hospital region (Appendix 1). Sociodemographic variables were self-reported. Additional covariates included clinical comorbidities and substance use as defined by validated ICD-10 code groupings published by the Healthcare Cost and Utilization Project in 2023 [25]. Descriptive statistics summarized sample characteristics. Categorical variables were reported as frequencies and percentages. Bivariate analyses and multivariable logistic regression were performed, with predictors tested for multicollinearity and interaction. Backward stepwise selection yielded the final model, adjusting for race, income, insurance, hospital characteristics, and substance use. All analyses incorporated NIS discharge weights, hospital clustering, and stratification variables to account for the complex survey design and to generate nationally representative estimates. Weighted adjusted odds ratios (AORs) with 95% confidence intervals are reported. Analyses were conducted using Stata SE 18.5 (College Station, TX, 2023), with statistical significance set at p < 0.05. Small cell sizes (< 10) were suppressed per data use agreements. This study was reviewed by the New York Medical College Institutional Review Board and determined to be exempt from full review because it used a publicly available, de-identified dataset (Protocol #23639). This study was conducted and reported in accordance with the STROBE (Strengthening the Reporting of Observational Studies in Epidemiology) guidelines (Appendix 2).

## Results

**Fig. 1:**
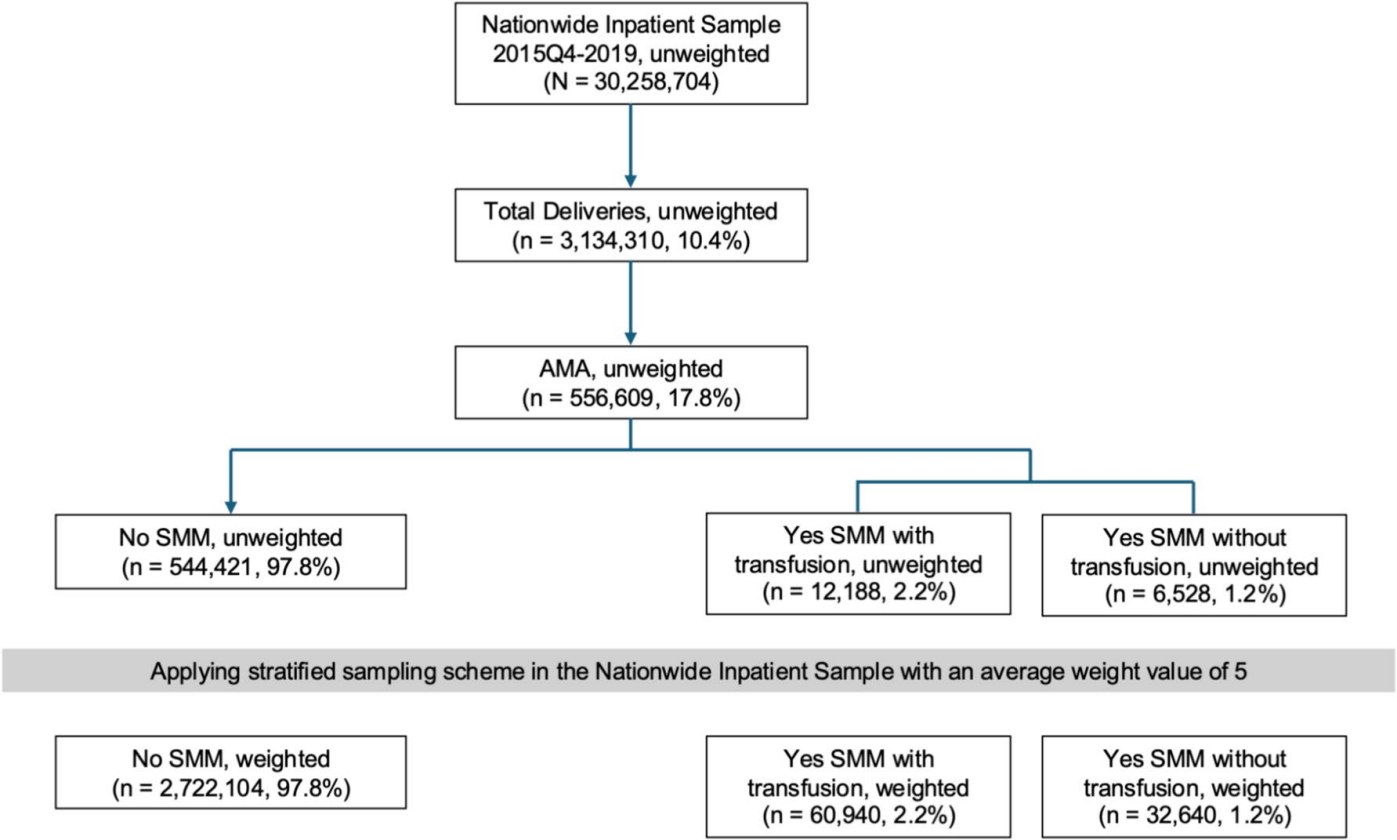
Flow diagram illustrating the selection of delivery hospitalizations for patients of advanced maternal age (AMA) from the Nationwide Inpatient Sample (NIS). Unweighted counts represent raw sample sizes, while weighted counts reflect national estimates based on stratified sampling with an average weight value of 5. The final analytic sample was used to evaluate the prevalence and predictors of severe maternal morbidity (SMM) with and without blood transfusion among patients of AMA

From Q4 2015 to 2019, the NIS database included 30,258,704 patients, of whom 3,134,310 (10.4%) had inpatient deliveries (Fig. 1). The final analytic sample consisted of 556,609 (17.8%) patients aged ≥ 35 years (AMA), with 12,188 (2.2%) experiencing SMM with blood transfusion and 6,528 (1.2%) experiencing nontransfusion SMM (Fig. 1). The most common SMM indicators were blood transfusion (1.29%), disseminated intravascular coagulation (0.29%), hysterectomy (0.28%), acute renal failure (0.20%), and acute respiratory distress syndrome (0.15%).

Most AMA deliveries occurred among patients aged 35–39 (81.8%), followed by 40–44 (17.0%) and ≥ 45 (1.2%). The cohort was predominantly White (52.8%), with Hispanic (19.6%), Black (12.1%), Asian/Pacific Islander (9.7%), and Native American & Other (5.8%) populations represented. Most patients were privately insured (65.0%), and nearly half (46.7%) were in the median income quartile. Deliveries were primarily at urban teaching hospitals (75.1%), with the South accounting for the largest regional share (34.8%) (Table 1).

**Table 1:** Descriptive Characteristics of Delivery Hospitalizations with Advanced Maternal Age—NIS 2015 Q4 to 2019 (Raw data and Weighted)

Total Study Population	556,609	2,783,044
	Unweighted *n* (%)	Weighted *n* (%)
Demographic Characteristics		
Age		
35—39	455,343 (81.81)	2,276,714 (81.81)
40—44	94,504 (16.98)	472,520 (16.98)
> 45	6,762 (1.21)	33,810 (1.21)
Race†		
Asian/PI	51,938 (9.73)	259,690 (9.73)
Black	64,680 (12.12)	323,400 (12.12)
Hispanic	104,558 (19.60)	522,790 (19.60)
NA & Other*	30,718 (5.76)	153,590 (5.76)
White	281,670 (52.79)	1,408,349 (52.79)
Income Quartile‡		
Median ($25,000-$44,999)	257,370 (46.71)	1,286,849 (46.71)
Low ($1-$24,999)	107,907 (19.58)	539,535 (19.58)
High ($45,000 or more)	185,746 (33.71)	928,730 (33.71)
Primary Payer§		
Private insurance	361,256 (64.03)	1,806,279 (65.03)
Public insurance**	164,205 (29.56)	821,025 (29.56)
Self-pay and Other	30,098 (5.42)	150,490 (5.42)
Hospital Characteristics		
Hospital Region		
South	193,489 (34.76)	967,445 (34.76)
Northeast	110,596 (19.87)	552,980 (19.87)
West	151,171 (27.16)	755,854 (27.16)
Midwest	101,353 (18.21)	506,765 (18.21)
Hospital Teaching Status		
Urban teaching	418,086 (75.11)	2,090,429 (75.11)
Urban non-teaching	109,368 (19.65)	546,840 (19.65)
Rural	29,155 (5.24)	145,775 (5.24)
Admission Year		
Year		
2019	133,977 (24.07)	669,885 (24.07)
2018	133,013 (23.90)	665,065 (23.90)
2017	130,082 (23.37)	650,409 (23.37)
2016	128,194 (23.03)	640,969 (23.03)
2015 Q4	31,343 (5.63)	156,715 (5.63)
Comorbidity (Prevalence)		
Obesity	59,477 (10.69)	297,385 (10.69)
High Blood Pressure	33,442 (6.01)	167,210 (6.01)
Type 2 Diabetes Mellitus	8,599 (1.54)	42,995 (1.54)
High Triglycerides	2,696 (0.48)	13,480 (0.48)
Low HDL Cholesterol	59 (0.01)	295 (0.01)
One MetS Condition	72,669 (13.06)	363,345 (13.06)
Two MetS Conditions	12,966 (2.33)	64,830 (2.33)
Metabolic Syndrome	1,846 (0.33)	9,230 (0.33)
Substance Use	25,670 (4.61)	128,350 (4.61)
Outcome (Prevalence)		
SMM without blood transfusion	6,528 (1.2)	32,640 (1.2)
SMM with blood transfusion	12,188 (2.2)	60,940 (2.2)

Comorbidities included obesity (10.7%), high blood pressure (6.0%), type 2 diabetes (1.5%), and MetS (0.3%) (Table 1). Bivariate analysis showed significantly higher SMM prevalence among patients with MetS (7.37% vs. 2.17% with transfusion and 5.36% vs. 1.16% nontransfusion) (Table 2). SMM prevalence increased significantly with advancing maternal age. Among patients aged 35–39, 40–44, and ≥ 45, rates of SMM with blood transfusion were 2.03%, 2.78%, and 4.67%, respectively, while rates of nontransfusion SMM were 1.07%, 1.53%, and 2.97% (Table 2).

**Table 2:** Bivariate analysis by severe maternal morbidity

Variables	SMM without Blood-transfusion			SMM with Blood-transfusion		
	No = 550,081	Yes = 6,528	*p*	No = 544,421	Yes = 12,188	*p*
	*n* (weighted %)	*n* (weighted %)		*n* (weighted %)	*n* (weighted %)	
Age			**< 0.001**			**< 0.001**
35—39	450,458 (98.93)	4,885 (1.07)		446,097 (97.97)	9,246 (2.03)	
40—44	93,062 (98.47)	1,442 (1.53)		91,878 (97.22)	2,626 (2.78)	
≥ 45	6,561 (97.03)	201 (2.97)		6,446 (95.33)	316 (4.67)	
Race			**< 0.001**			**< 0.001**
White	278,888 (99.01)	2,782 (0.99)		276,734 (98.25)	4,936 (1.75)	
Black	63,400 (98.02)	1,280 (1.98)		62,260 (96.26)	2,420 (3.74)	
Hispanic	103,348 (98.84)	1,210 (1.16)		102,146 (97.69)	2,412 (2.31)	
Asian/PI	51,289 (98.75)	649 (1.25)		50,686 (97.59)	1,252 (2.41)	
NA & Other*	30,340 (98.77)	378 (1.23)		29,981 (97.60)	737 (2.40)	
Income Quartile			**< 0.001**			**< 0.001**
Median ($25,000-$44,999)	106,332 (98.54)	1,575 (1.46)		251,841 (97.85)	5,529 (2.15)	
Low ($1-$24,999)	254,363 (98.83)	3,007 (1.17)		104,935 (97.25)	2,972 (2.75)	
High ($45,000 or more)	183,880 (99.00)	1,866 (1.00)		182,208 (98.10)	3,538 (1.90)	
Primary Payer			**< 0.001**			**< 0.001**
Private insurance	357,536 (98.97)	3,720 (1.03)		354,333 (98.08)	6,923 (1.92)	
Public insurance**	161,724 (98.49)	2,481 (1.51)		159,565 (97.17)	4,640 (2.83)	
Self-pay and Other	29,781 (98.95)	317 (1.05)		29,493 (97.99)	605 (2.01)	
Hospital Region			0.533			**< 0.001**
South	109,265 (98.80)	1,331 (1.20)		189,193 (97.78)	4,296 (2.22)	
Northeast	100,170 (98.83)	1,183 (1.17)		107,776 (97.45)	2,820 (2.55)	
West	191,268 (98.85)	2,221 (1.15)		147,964 (97.88)	3,207 (2.12)	
Midwest	149,378 (98.81)	1,793 (1.19)		99,488 (98.16)	1,865 (1.84)	
Hospital Teaching Status			**< 0.001**			**< 0.001**
Urban teaching	412,683 (98.71)	5,403 (1.29)		408,302 (97.66)	9,784 (2.34)	
Urban non-teaching	108,471 (99.18)	897 (0.82)		107,536 (98.32)	1,832 (1.68)	
Rural	28,927 (99.22)	228 (0.78)		28,583 (98.04)	572 (1.96)	
Metabolic Syndrome			**< 0.001**			** < 0.001**
No	548,334 (98.84)	6,429 (1.16)		542,711 (97.83)	12,052 (2.17)	
Yes	1,747 (94.64)	99 (5.36)		1,710 (92.63)	136 (7.37)	
Substance Use			**< 0.001**			**< 0.001**
No	525,004 (98.88)	5,935 (1.12)		519,732 (97.89)	11,207 (2.11)	
Yes	25,077 (97.69)	593 (2.31)		24,689 (96.18)	981 (3.82)	

Table 1**.** Descriptive characteristics of delivery hospitalizations among patients of advanced maternal age in the United States, Nationwide Inpatient Sample (2015 Q4–2019). Data are presented as unweighted counts (%) and weighted counts (%). Comorbidity and outcome statistics reflect the prevalence of each condition. Percentages are calculated excluding missing values. †Race had 4.14% missing values; ‡Income quartile had 1.00% missing values; §Primary payer had 0.19% missing values. *Native American was combined with “Other” due to small sample size. **Public insurance includes Medicare and Medicaid. Metabolic syndrome (MetS): defined as ≥ 3 of 5 conditions: obesity, hypertension, type 2 diabetes, low HDL, and hypertriglyceridemia.

Table 2**.** Bivariate analysis of demographic, clinical, and hospital characteristics by severe maternal morbidity (SMM) with and without blood transfusion among delivery hospitalizations of patients with advanced maternal age, Nationwide Inpatient Sample (2015 Q4–2019). Data are presented as weighted percentages with p-values for comparisons across SMM groups. *Native American was combined with “Other” due to small sample size. **Public insurance includes Medicare and Medicaid.

Table 3 Adjusted odds ratios (aORs) with 95% confidence intervals (CIs) for predictors of severe maternal morbidity (SMM) with and without blood transfusion among delivery hospitalizations of patients with advanced maternal age, Nationwide Inpatient Sample (2015 Q4–2019). aORs were derived from weighted survey logistic regression models, adjusted for demographic, clinical, and hospital covariates. Statistical significance was defined as p < 0.05, < 0.01, < 0.001.

**Table 3:** Adjusted Odds Ratios (with 95% Confidence Intervals) for Predictors of Severe Maternal Morbidity (SMM with and without blood transfusion) in a Weighted Survey Logistic Regression Analysis

Predictors	SMM without Blood-transfusion		SMM with Blood-transfusion	
	aOR (95% CI)	*p*	aOR (95% CI)	*p*
Metabolic Syndrome				
No	(Ref)		(Ref)	
Yes	3.59 (2.90–4.44)	** < 0.001**	2.75 (2.29–3.30)	** < 0.001**
Advanced Maternal Age				
35—39	(Ref)		(Ref)	
40—44	1.38 (1.29–1.46)	** < 0.001**	1.32 (1.26–1.38)	** < 0.001**
≥ 45	2.81 (2.41–3.27)	** < 0.001**	2.31 (2.05–2.61)	** < 0.001**
Race				
White	(Ref)		(Ref)	
Black	1.74 (1.62–1.87)	** < 0.001**	1.91 (1.81–2.02)	** < 0.001**
Hispanic	1.06 (0.98–1.15)	0.125	1.21 (1.14–1.29)	** < 0.001**
Asian/PI	1.28 (1.17–1.40)	** < 0.001**	1.41 (1.32–1.50)	** < 0.001**
NA & Other*	1.18 (1.05–1.32)	** < 0.01**	1.28 (1.18–1.39)	** < 0.001**
Income Quartile				
Median ($25,000-$44,999)	(Ref)		(Ref)	
Low ($1-$24,999)	1.09 (1.02–1.17)	** < 0.01**	1.10 (1.05–1.16)	** < 0.001**
High ($45,000 or more)	0.91 (0.85–0.97)	** < 0.01**	0.94 (0.89–0.98)	** < 0.01**
Primary Payer				
Private insurance	(Ref)		(Ref)	
Public insurance**	1.21 (1.14–1.29)	** < 0.001**	1.20 (1.15–1.25)	** < 0.001**
Self-pay and Other	1.00 (0.88–1.13)	0.977	1.00 (0.91–1.09)	0.927
Hospital Teaching Status				
Urban teaching	(Ref)		(Ref)	
Urban non-teaching	0.66 (0.61–0.72)	** < 0.001**	0.76 (0.71–0.80)	** < 0.001**
Rural	0.58 (0.50–0.67)	** < 0.001**	0.88 (0.80–0.97)	** < 0.05**
Hospital Region				
South	(Ref)		(Ref)	
Northeast	1.07 (0.98–1.16)	0.116	1.21 (1.13–1.29)	** < 0.001**
West	1.15 (1.06–1.24)	** < 0.001**	1.06 (0.99–1.13)	0.1
Midwest	1.11 (1.03–1.21)	** < 0.01**	0.90 (0.84–0.96)	** < 0.01**
Substance Use				
No	(Ref)		(Ref)	
Yes	1.85 (1.69–2.03)	** < 0.001**	1.68 (1.56–1.81)	** < 0.001**

**Fig. 2:**
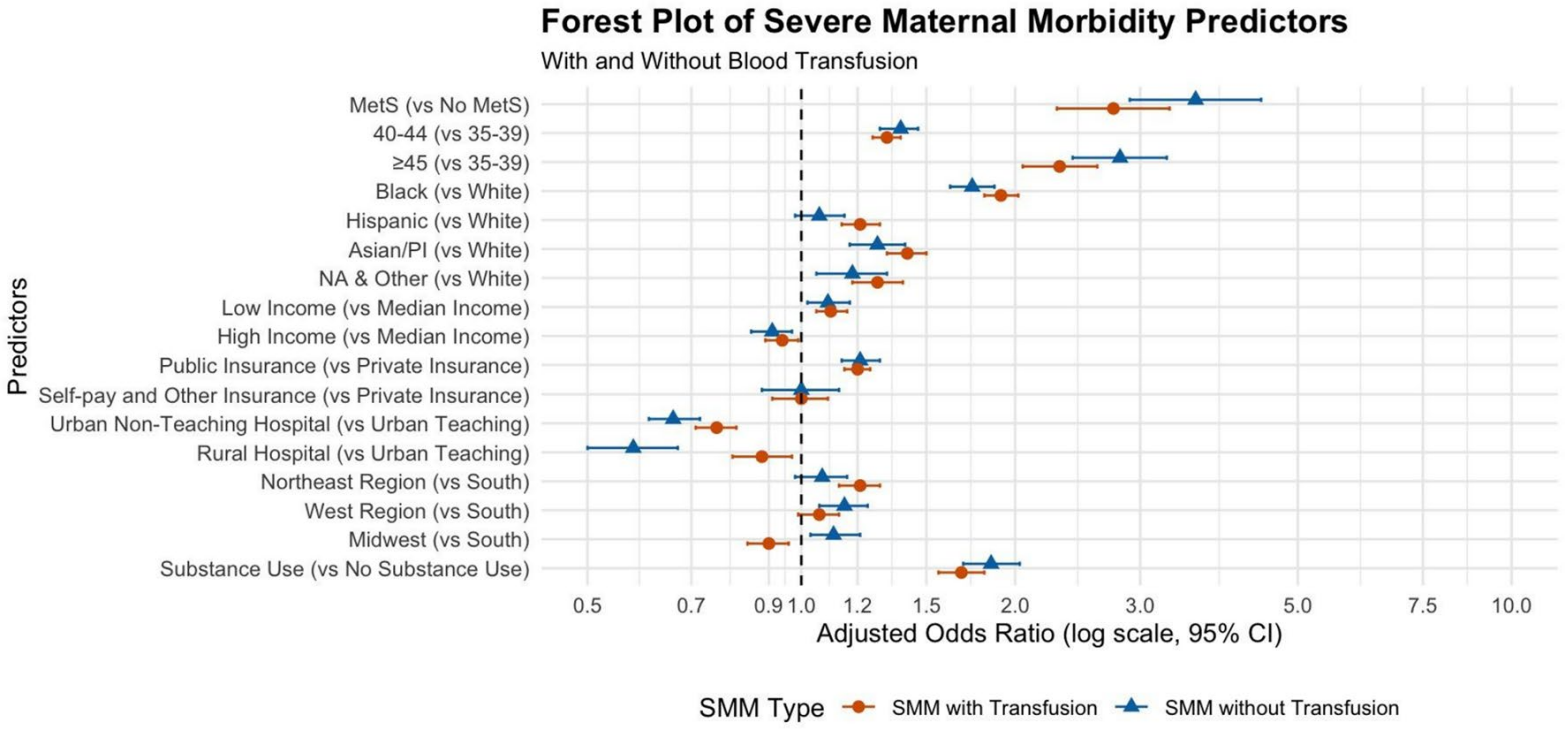
Forest plot illustrating the adjusted odds ratios (OR) with 95% confidence intervals (CI) for predictors of severe maternal morbidity (SMM) with (orange) and without (blue) blood transfusion among individuals of advanced maternal age (AMA). The x-axis represents the adjusted OR using a log scale, with the dashed vertical line at 1 indicating no effect. Predictors include maternal metabolic syndrome (MetS), age groups, race/ethnicity, socioeconomic status (income and insurance type), hospital characteristics, geographic region, and substance use

Adjusted odds ratios (AORs) for SMM predictors are presented in Table 3 and represented in Fig. 2. The final model included MetS, age, race, income quartile, insurance type, hospital teaching status, region, and substance use. MetS was the strongest predictor of SMM, associated with a nearly threefold increase in SMM with blood transfusion (AOR 2.75, 95% CI 2.29–3.30, p < 0.001) and a more than threefold increase in nontransfusion SMM (AOR 3.59, 95% CI 2.90–4.44, p < 0.001) (Table 3; Fig. 2). Additionally, higher AMA subgroups had greater SMM risk. Patients aged 40–44 had 32% higher odds (AOR 1.32, 95% CI: 1.26–1.38), and those ≥ 45 had over twice the odds of SMM with blood transfusions compared to ages 35–39 (AOR 2.31, 95% CI: 2.05–2.61). The trends were similar with slightly higher odds of nontransfusion SMM (Table 3; Fig. 2). Associations between SMM and race, socioeconomic factors, hospital characteristics, and geographic region are summarized in Table 3 and Fig. 2.

## Discussion

Among pregnant patients of AMA, MetS was the strongest independent predictor of SMM, nearly tripling the risk, with a greater effect on nontransfusion severe maternal morbidity (AOR 3.59, 95% CI 2.90–4.44) than on morbidity with transfusion (AOR 2.75, 95% CI 2.29–3.30). The risk of SMM also increased with advancing age: patients aged 40–44 had a 32% higher risk than those 35–39, and patients 45 and older had more than double the risk. These trends were consistent for nontransfusion severe maternal morbidity.

We investigated SMM outcomes in pregnant patients of AMA with MetS and the associations with various demographic and health risk factors. In our study, 1,846 (0.3%) pregnant patients had MetS as defined by ICD-10 codes at hospital delivery, contrasting with global estimates of 15–18% depending on definitions used [18]. This large discrepancy likely reflects both differing diagnostic criteria and underreporting via ICD-10 codes, particularly at delivery. Therefore, our findings likely underestimate the true prevalence of MetS in AMA patients in the U.S., for which no national estimate currently exists. Similarly, obesity prevalence in our sample (10.7%) was lower than national estimates (20–30%), consistent with undercoding in administrative datasets [26, 27]. National data show that obesity rates among women entering pregnancy are comparable across age groups, including advanced maternal age, with about 29.5% classified as obese in 2020; while normal BMI declines with age, obesity prevalence remains stable or slightly higher in older women [28].

MetS emerged as the strongest independent predictor, nearly tripling the risk of both transfusion and nontransfusion SMM. Notably, the association was stronger for nontransfusion SMM (AOR 3.59, 2.90–4.44, p < 0.001) compared to SMM with transfusion (AOR 2.75, 2.29–3.30, p < 0.001), suggesting that MetS has a greater impact on the more severe morbidity outcomes. While prior studies have examined individual comorbidities like obesity, diabetes, and hypertension, few have considered MetS as a combined entity in AMA [29, 30]. Furthermore, these studies analyzed the impact of comorbidities throughout the pregnancy, rather than at hospital delivery like our study. Though the term MetS was introduced in 1998, it remains underused clinically despite the common co-occurrence of its components [31]. This is the first population-level study to quantify the impact of MetS on SMM in AMA patients, underscoring the need for early recognition and management.

We also found that the risk of SMM rises progressively with age. Patients aged 40–44 had a 32% higher risk of SMM than those aged 35–39, and those ≥ 45 had more than double the risk. These findings were similar for nontransfusion SMM and aligned with prior studies showing age-associated increases in SMM [14, 32]. Notably, our study population is older than in previous research, focusing on an AMA cohort, and our findings reflect population-level data in the US. 

Our study also reveals stark racial disparities in SMM among AMA patients. Black patients had nearly double the risk compared to White patients, with elevated risks also observed among Asian/Pacific Islander, Native American, and Hispanic patients. These results are consistent with prior national studies showing higher SMM rates among ethnic minorities, often attributed to differences in hypertensive disorders, access to care, and systemic inequities, persisting across age groups [33–36].

Insurance and income status also affected outcomes. Patients with public insurance had a 20% higher risk of SMM with and without blood transfusion compared to those with private coverage. Similarly, patients in the low-income group had a 10% increased risk relative to the median income group. These findings are in line with earlier research and reflect broader socioeconomic barriers to optimal maternal care [36, 37].

Interestingly, SMM risk was higher in urban teaching hospitals than in urban non-teaching or rural hospitals. This diverges from previous research showing worse outcomes in rural settings [38]. However, teaching hospitals often manage higher-risk pregnancies, which could explain the discrepancy. Regionally, the Northeast had higher SMM with blood transfusion, while the Midwest and West had increased nontransfusion SMM, suggesting potential differences in transfusion use, morbidity prevalence, and documentation.

Substance use was associated with a 68% higher risk of SMM with blood transfusion, consistent with prior research showing links to preeclampsia, preterm labor, and other complications [39]. Another study using the NIS database found a 17% increase in SMM among patients over age 34 with opioid or stimulant use, supporting our findings [40].

MetS is a powerful yet underrecognized driver of SMM in women of AMA. The nearly threefold increase in risk, particularly for nontransfusion outcomes, suggests that combined obesity, hypertension, and diabetes markedly heighten vulnerability to pregnancy complications. These findings underscore the need for preconception and prenatal screening for MetS components and proactive management of blood pressure, glycemic control, and weight. Early identification of AMA patients with MetS should prompt enhanced monitoring, potential referral to tertiary centers, and individualized counseling.

The age-related rise in SMM, alongside persistent racial and socioeconomic disparities, highlights systemic gaps in maternal care. Policymakers should prioritize access to comprehensive preconception and prenatal services for at-risk and marginalized populations. Hospital systems, particularly urban teaching centers with higher morbidity rates, should evaluate care pathways, transfusion practices, and resource allocation to mitigate preventable complications.

Future research should elucidate mechanisms linking MetS to SMM in AMA, whether through vascular dysfunction, inflammation, or synergistic metabolic effects. Prospective cohorts spanning preconception to postpartum are needed to define temporal relationships and intervention windows beyond delivery-only datasets.

Improved data capture, including validated ICD-10 coding for MetS and integration of laboratory measures (lipids, glucose, BMI, blood pressure), would refine prevalence estimates and risk characterization. Interventional and implementation studies should assess whether optimizing metabolic health before or early in pregnancy reduces SMM risk, while addressing social determinants, systemic racism, and care access. Additionally, labor and delivery units may benefit from developing risk stratification protocols to identify AMA patients with MetS and other high-risk characteristics, allowing for enhanced surveillance, multidisciplinary planning, and hemorrhage preparedness. Future research should evaluate whether implementation of such risk stratification tools reduces rates of severe maternal morbidity. Health system interventions, such as enhanced preconception counseling, early referral pathways, and quality improvement initiatives in high-volume centers, warrant evaluation for scalable risk-reduction strategies.

Our study has notable strengths. First, this study addresses an important and timely clinical question given the increasing prevalence of AMA pregnancies and metabolic comorbidities.

Second, the NIS is the largest hospital care dataset in the U.S., enhancing statistical power and generalizability. It includes a diverse population across racial, socioeconomic, and geographic groups, making our findings widely applicable. This scope allows for robust public health insights not achievable in smaller, single-center studies to study rare conditions like MetS. Finally, differentiation of SMM with and without blood transfusion provides nuanced insight into morbidity severity.

This study has several limitations. The NIS captures data only at delivery, lacking longitudinal information on maternal health, treatment adherence, and disease control, which may introduce unmeasured confounding. Reliance on ICD-10 codes limits accuracy and may lead to underreporting or misclassification, potentially underestimating MetS and related conditions [31]. For instance, obesity was identified through diagnostic codes rather than BMI, likely underestimating its prevalence; however, this approach has been validated and remains standard in population-level research [26, 41]. Additionally, our definition of MetS was restricted to chronic conditions present prior to pregnancy and did not include pregnancy-related diagnoses such as gestational diabetes or hypertensive disorders of pregnancy. This approach was intended to capture baseline metabolic health rather than conditions that may arise during pregnancy and lie along the causal pathway to SMM. However, this may underestimate the overall burden of metabolic dysfunction in this population and may limit our ability to assess the contribution of gestational metabolic complications to SMM risk. Finally, parity, a key determinant of obstetric outcomes and potential confounder for SMM, is not available in the NIS, precluding direct adjustment. Because women of advanced maternal age are more often multiparous, this may attenuate but not eliminate residual confounding related to parity [7].

## Conclusion

MetS was strongly associated with an increased risk of SMM among patients with AMA in this nationally representative cohort. Future research should refine screening and intervention strategies for high-risk AMA patients, particularly those with MetS, those with substance use, and those from marginalized backgrounds. Longitudinal studies are needed to explore how maternal health evolves throughout pregnancy. National initiatives should also focus on expanding preconception care, enhancing screening protocols for MetS, and addressing racial and socioeconomic disparities through targeted quality improvement efforts.

## Appendix 1

Pregnancy Status, Delivery, Procedure, and Exposure Variable ICD-10 Codes.

**Table:** 

Category	ICD-10 Code(s)
Delivery Diagnosis	Z37.0–Z37.1, Z37.2–Z37.7, Z37.51–Z37.54, Z37.59, Z37.60–Z37.64, Z37.69, Z37.9
Delivery Procedure	10E0XZZ, 10D00Z0–10D00Z2, 10D07Z3–10D07Z8
High Blood Pressure	I10–I16*
Altered Fasting Glucose (Diabetes Mellitus)	E11*
BMI ≥ 30 (Obesity)	E66.0–E66.2, E66.8, E66.9, Z68.3–Z68.4
Low HDL Cholesterol	E78.6, E88.1
High Triglycerides	E78.1, E78.2, E78.3, E78.4
Substance Use	F10/F19, O99.31–O99.315, O99.32–O99.325, O99.33–O99.335

SMM Indicators and Corresponding ICD-10 Codes.

**Table:** 

Severe Maternal Morbidity Indicator	ICD-10 Code(s)
Acute Myocardial Infarction	I2101-I2102, I2109, I2111, I2119, I2121, I2129, I213-I214, I219, I21A1, I21A9, I21B, I220-I222, I228-I229
Aneurysm	I71.00–I71.9, I79.0
Acute Renal Failure	N17.0–N17.9, O90.4
Acute Respiratory Distress Syndrome	J80, J95.1-J95.3, J95.821-J95.822, J96.00-J96.02, J96.20-J96.22, J96.90-J96.92, R06.03, R09.2
Amniotic Fluid Embolism	O88.112-O88.113, O88.119, O88.12-O88.13
Cardiac Arrest / Ventricular Fibrillation	I46.2, I46.8-I46.9, I49.01-I49.02, I49.1-I49.3
Disseminated Intravascular Coagulation	D65, D68.8-D68.9, O45.002-O45.003, O45.009, O45.012-O45.013, O45.019, O45.022-O45.023, O45.029, O45.092-O45.093, O45.099, O46.002-O46.003, O46.009, O46.012-O46.013, O46.019, O46.022-O46.023, O46.029, O46.092-O46.093, O46.099, O67.0, O72.3
Eclampsia	O15.00, O15.02-O15.03, O15.1-O15.2, O15.9
Heart Failure / Arrest During Surgery or Procedure	I97.120-I97.121, I97.130-I97.131, I97.710-I97.711
Puerperal Cerebrovascular Disorders	I60.32, I60.4–I60.9, I61.0–I61.6, I61.8–I61.9, I62.00–I62.03, I62.1, I62.9, I63.00–I63.03, I63.1–I63.59, I63.6, I63.8, I63.81, I63.89, I63.9, I65.0–I65.03, I65.09, I65.1, I65.2, I65.21–I65.23, I65.29, I65.8, I65.9, I66, I66.0–I66.03, I66.09, I66.1–I66.13, I66.19, I66.2–I66.23, I66.29, I66.3, I66.8, I66.9, I67, I67.0–I67.7, I67.8, I67.81–I67.85, I67.850, I67.858, I67.89, I67.9, I68, I68.0, I68.2, I68.8, O22.50, O22.52, O22.53, I97.810, I97.811, I97.820, I97.821, O87.3
Pulmonary Edema / Acute Heart Failure	I50.1, I50.20–I50.21, I50.23, I50.30–I50.31, I50.33, I50.40–I50.41, I50.43, I50.810–I50.811, I50.813–I50.814, I50.82–I50.84, I50.89, I50.9, J81.0
Severe Anesthesia Complications	O29.112–O29.119, O29.122–O29.129, O29.192–O29.199, O29.212–O29.219, O29.292–O29.299, O74.0–O74.3, O89.01, O89.09, O89.1–O89.2, T88.2XXA, T88.3XXA
Sepsis	A32.7, A40.0–A40.1, A40.3, A40.8–A40.9, A41.0–A41.5, A41.50–A41.54, A41.59, A41.8, A41.81, A41.89–A41.9, I76, O85, O86.04, R65.20–R65.21, T81.12XA, T81.44XA
Shock	O75.1, R57.0–R57.1, R57.8–R57.9, T78.2XXA, T81.10XA–T81.11XA, T81.19XA, T88.6XXA
Sickle Cell Disease with Crisis	D57.00–D57.02, D57.211–D57.219, D57.411–D57.419, D57.811–D57.819
Air and Thrombotic Embolism	I26.0–I26.02, I26.09, I26.9, I26.90, I26.92–I26.94, I26.99, O88.012–O88.03, O88.212–O88.23, O88.312–O88.33, O88.812–O88.83, T80.0XXA
Conversion of Cardiac Rhythm	5A12012, 5A2204Z
Blood Transfusion	30230H0, 30230K0, 30230L0, 30230M0, 30230N0, 30230P0, 30230R0, 30230T0, 30230H1, 30230K1, 30230L1, 30230M1, 30230N1, 30230P1, 30230R1, 30230T1, 30233H0, 30233K0, 30233L0, 30233M0, 30233N0, 30233P0, 30233R0, 30233T0, 30233H1, 30233K1, 30233L1, 30233M1, 30233N1, 30233P1, 30233R1, 30233T1, 30240H0, 30240K0, 30240L0, 30240M0, 30240N0, 30240P0, 30240R0, 30240T0, 30240H1, 30240K1, 30240L1, 30240M1, 30240N1, 30240P1, 30240R1, 30240T1, 30243H0, 30243K0, 30243L0, 30243M0, 30243N0, 30243P0, 30243R0, 30243T0, 30243H1, 30243K1, 30243L1, 30243M1, 30243N1, 30243P1, 30243R1, 30243T1
Hysterectomy	0UT90ZL, 0UT90ZZ, 0UT97ZL, 0UT97ZZ
Temporary Tracheostomy	0B110F4, 0B113F4, 0B114F4
Ventilation	5A1935Z, 5A1945Z, 5A1955Z

NIS Database Variables.

**Table:** 

Variable	NIS Variable Name	Variable Categories
Race	RACE, RACE_X	White, Black, Hispanic, Asian/PI, NA & Other*, Missing
Primary Payer	PAY1, PAY1_X	Private insurance, Public insurance**, Self-pay and Other, Missing
Income Quartile	ZIPINC_QRTL	Median ($25,000–$44,999), Low ($1–$24,999), High ($45,000 or more), Missing
Hospital Region	HOSP_REGION	South, Northeast, West, Midwest
Hospital Teaching	HOSP_TEACH	Urban teaching, Urban non-teaching, Rural

* ‘Native American (NA)’ category was merged with ‘Other’ due to small sample size for improved analysis.

## Appendix 2

STROBE Statement—Checklist of items that should be included in reports of cross-sectional studies.

**Table:** 

Item	No	Recommendation	Page No
**Title and abstract**			
	1(a)	Indicate the study’s design with a commonly used term in the title or the abstract	Title page; Abstract (p.1–2)
	1(b)	Provide in the abstract an informative and balanced summary of what was done and what was found	Abstract (p.1–2)
**Introduction**			
Background/rationale	2	Explain the scientific background and rationale for the investigation being reported	Introduction (p.2–3)
Objectives	3	State specific objectives, including any prespecified hypotheses	End of Introduction (p.3)
**Methods**			
Study design	4	Present key elements of study design early in the paper	Methods, first paragraph (p.3–4)
Setting	5	Describe the setting, locations, and relevant dates	Methods, NIS description (p.3–4)
Participants	6(a)	Give eligibility criteria and selection of participants	Methods, study population (p.3–4)
Variables	7	Define outcomes, exposures, confounders, diagnostic criteria	Methods (p.4–5)
Data sources/measurement	8*	Sources of data and measurement methods	Methods + Appendix 1 (p.3–5)
Bias	9	Describe efforts to address bias	Methods (design limitations); Discussion limitations (p.4, p.12–13)
Study size	10	Explain how study size was determined	Results (flow diagram + NIS sample) (p.5–6)
Quantitative variables	11	Explain handling of quantitative variables	Methods (age groupings, categorization) (p.4–5)
Statistical methods	12(a)	Describe statistical methods and confounding control	Methods (p.5)
	12(b)	Subgroup/interactions	Methods + Results (age strata, SMM definitions) (p.5–6, p.7–9)
	12(c)	Missing data handling	Table 1 footnote + Methods (p.5–6)
	12(d)	Sampling strategy accounted for	Methods (NIS weighting, clustering) (p.5)
	12(e)	Sensitivity analyses	Methods/Results (SMM with vs without transfusion) (p.4–6)
**Results**			
Participants	13(a)	Numbers at each stage	Results + Fig. 1 (p.5–6)
	13(b)	Reasons for non-participation	Not applicable (administrative dataset)
	13(c)	Flow diagram	Figure 1 (p.5–6)
Descriptive data	14(a)	Participant characteristics	Table 1 (p.6–7)
	14(b)	Missing data	Table 1 footnote (p.6–7)
Outcome data	15	Outcome events and summary measures	Results + Table 2 (p.6–8)
Main results	16(a)	Unadjusted and adjusted estimates with CIs	Table 3 + Fig. 2 (p.8–10)
	16(b)	Category boundaries	Methods (age, income categories) (p.4–5)
	16(c)	Absolute risk translation	Reported as prevalence percentages (p.6–8)
Other analyses	17	Subgroups and sensitivity analyses	Results (age strata, transfusion vs nontransfusion SMM) (p.6–9)
**Discussion**			
Key results	18	Summarize key results	First paragraph Discussion (p.10)
Limitations	19	Discuss limitations and bias	Discussion limitations (p.12–13)
Interpretation	20	Overall interpretation	Discussion (p.10–12)
Generalizability	21	External validity	Discussion (NIS national dataset) (p.12)
**Other information**			
Funding	22	Source of funding	Title page / declarations (no funding)

## Data Availability

The data used in this study were obtained from the Nationwide Inpatient Sample (NIS), part of the Healthcare Cost and Utilization Project (HCUP), Agency for Healthcare Research and Quality. The NIS is a publicly available, de-identified database that can be accessed by researchers who complete the HCUP Data Use Agreement and purchase the data from the HCUP Central Distributor (https://www.hcup-us.ahrq.gov/). Due to data use restrictions, the authors are not permitted to share the data directly. All analyses were conducted in accordance with HCUP guidelines.
